# Long-Term Effects in Bone Mineral Density after Different Bariatric Procedures in Patients with Type 2 Diabetes: Outcomes of a Randomized Clinical Trial

**DOI:** 10.3390/jcm9061830

**Published:** 2020-06-11

**Authors:** Fernando Guerrero-Pérez, Anna Casajoana, Carmen Gómez-Vaquero, Nuria Virgili, Rafael López-Urdiales, Laura Hernández-Montoliu, Jordi Pujol-Gebelli, Javier Osorio, Anna Prats, Anna Vidal-Alabró, Manuel Pérez-Maraver, Sonia Fernández-Veledo, Joan Vendrell, Nuria Vilarrasa

**Affiliations:** 1 Department of Endocrinology and Nutrition, Bellvitge University Hospital-IDIBELL, L’Hospitalet de Llobregat, 08907 Barcelona, Spain; fguerrerop@bellvitgehospital.cat (F.G.-P.); mvirgili@bellvitgehospital.cat (N.V.); rafaellopez@bellvitgehospital.cat (R.L.-U.); laura.hermont@gmail.com (L.H.-M.); mmperez@bellvitgehospital.cat (M.P.-M.); 2 Bariatric Surgery Unit, Bellvitge University Hospital-IDIBELL, L’Hospitalet de Llobregat, 08907 Barcelona, Spain; acbadia@bellvitgehospital.cat (A.C.); jpujol@bellvitgehospital.cat (J.P.-G.); josorio@bellvitgehospital.cat (J.O.); 3 Department of Rheumatology, Bellvitge University Hospital-IDIBELL, L’Hospitalet de Llobregat, 08907 Barcelona, Spain; cgomezvaq@ambitcp.catsalut.net; 4 Clinical Nutrition Unit, Bellvitge University Hospital-IDIBELL, L’Hospitalet de Llobregat, 08907 Barcelona, Spain; apratsf@bellvitgehospital.cat; 5 Instituto de Investigación Biomédica-IDIBELL, L’Hospitalet de Llobregat, 08907 Barcelona, Spain; avidala@idibell.cat; 6 CIBERDEM-CIBER de Diabetes y Enfermedades Metabólicas Asociadas, Instituto de Salud Carlos III, 28014 Madrid, Spain; sonia.fernandezveledo@gmail.com (S.F.-V.); jvortega2002@gmail.com (J.V.); 7 Pere Virgili Research Institute (IISPV), University Hospital Joan XXIII, 43005and Rovira i Virgili University, 43003 Tarragona, Spain

**Keywords:** bone mineral density, bariatric surgery, gastrointestinal hormones

## Abstract

There is scant evidence of the long-term effects of bariatric surgery on bone mineral density (BMD). We compared BMD changes in patients with severe obesity and type 2 diabetes (T2D) 5 years after randomization to metabolic gastric bypass (mRYGB), sleeve gastrectomy (SG) and greater curvature plication (GCP). We studied the influence of first year gastrointestinal hormone changes on final bone outcomes. Forty-five patients, averaging 49.4 (7.8) years old and body mass index (BMI) 39.4 (1.9) kg/m^2^, were included. BMD at lumbar spine (LS) was lower after mRYGB compared to SG and GCP: 0.89 [0.82;0.94] vs. 1.04 [0.91;1.16] vs. 0.99 [0.89;1.12], *p* = 0.020. A higher percentage of LS osteopenia was present after mRYGB 78.6% vs. 33.3% vs. 50.0%, respectively. BMD reduction was greater in T2D remitters vs. non-remitters. Weight at fifth year predicted BMD changes at the femoral neck (FN) (adjusted *R*^2^: 0.3218; *p* = 0.002), and type of surgery (mRYGB) and menopause predicted BMD changes at LS (adjusted *R*^2^: 0.2507; *p* < 0.015). In conclusion, mRYGB produces higher deleterious effects on bone at LS compared to SG and GCP in the long-term. Women in menopause undergoing mRYGB are at highest risk of bone deterioration. Gastrointestinal hormone changes after surgery do not play a major role in BMD outcomes.

## 1. Introduction

Bariatric surgery has become an increasingly common treatment for severe obesity due to its outstanding results in long-term weight loss and sustained improvement in obesity-related comorbidities, mortality and quality of life [1,2]. However, there is arising evidence of negative effects on bone health and risk of bone fractures at long-term in patients with surgically induced weight loss [3,4,5]. Patients with obesity and type 2 diabetes (T2D), before undergoing bariatric surgery, could have an increased risk of bone fracture. Contrary to what was believed, although obese individuals usually have higher bone mineral density (BMD) compared to non-obese individuals [6,7], obesity per se is not protective and there is a site- and gender-specific relationship between body mass index (BMI) and fracture risk [8,9,10,11]. On the other hand, individuals with T2D have normal or higher BMD in comparison with those without diabetes [12,13], but their bone quality is diminished and their risk of fracture is also increased [14]. Hence, patients with obesity and T2D whom undergo bariatric surgery meet many potentially deleterious factors on bone health that should be taken into consideration.

The negative skeletal effects of bariatric surgery are multifactorial and probably procedure-specific [15]. Several mechanisms seem to be involved in postoperative bone loss, including mechanical unloading induced by weight reduction and nutrient deficiencies, such as protein, calcium, vitamin D and the subsequent secondary hyperparathyroidism [15,16]. Furthermore, gastrointestinal hormonal changes caused by anatomical shift and weight loss could affect bone health [16,17]. Data coming from animal models [18,19,20] and a few human studies [21,22] have shown a relationship between changes in gastric inhibitory polypeptide (GIP), ghrelin, glucagon-like peptide-1 (GLP-1) or peptide YY (PYY) and changes in bone remodeling markers. Other factors like adipokines (leptin, adiponectin), muscle mass loss and bone marrow fat could be implicated [23,24,25,26]. All these modifications and their impact on bone might vary between different surgical procedures and are probably more pronounced after malabsorptive or, more accurately named, hypoabsortive techniques [15,17,27]. Some studies have compared BMD outcomes after different surgical procedures, although most of them in a non-randomized manner and at short-term (1–3 years of follow-up) [21,24,28,29,30,31,32].

Our aim in the present study was to compare BMD changes in patients with obesity and T2D, 5 years after being randomized to: metabolic gastric bypass (mRYGB), sleeve gastrectomy (SG), and greater curvature plication (GCP) in the setting of a randomized controlled trial (RCT). We have also analyzed the relationship between changes in gastrointestinal hormones during the first year after surgery with 5 years skeletal outcome.

## 2. Materials and Methods

This research was part of a prospective, single center and non-blinded RCT, including patients with T2D and obesity. Participants were consecutively recruited from a morbid obesity outpatient clinic. Inclusion criteria were as follows: age between 18 and 60 years, BMI 35–43 kg/m^2^, T2D on hypoglycemic agents alone, insulin or both. Exclusion criteria were type 1 diabetes or positivity for GAD autoantibodies, secondary forms of diabetes, acute metabolic complications, liver disease, renal dysfunction or patients under anticoagulant treatment, previous bariatric surgery, congenital or acquired abnormalities of the digestive tract, pregnancy, nursing or desired pregnancy in the 12 months following inclusion, and corticoid use by the oral or intravenous route for more than 14 consecutive days in the last three months.

The study protocol was previously published [33]. We used Buse criteria to define T2D remission at 5 year of follow-up [34]. There were no changes to methods after the commencement of the study. The study was carried out according to the principles of the Declaration of Helsinki, and all patients signed an informed consent. This manuscript has been approved for its publication by the Research Ethics Committee of our institution (reference PR144/20). The trial was registered at www.controlledtrials.com as ISRCTN14104758.

### 2.1. Randomization

The randomization process was performed by the statistic department using a computer software program that generated the random sequence. The allocation of patients was assigned by simple randomization 1:1:1 to undergo mRYGB, SG or GCP, using opaque sealed sequentially numbered envelopes with stratification according to baseline levels of HbA1c (greater or lower/equal to 7%). After signing informed consent, patients were allocated to a specific surgery. The study was therefore not blinded and the patients, endocrinologist and surgeon were informed about the type of surgery procedure the patient had been allocated to. After surgical intervention, a multivitamin pill once daily and calcium/vitamin D (1000 mg/800 IU) was prescribed in all participants. In addition, patients undergoing mRYGB received 16.000 IU of vitamin D every 15 days. All patients were managed by the same endocrinologist and two dietitians. They were given the same diet, physical activity and behavioral counseling during the follow-up.

### 2.2. Anthropometric Parameters

Weight change after surgery was referred to as total weight loss percentage (TWL%). Body composition (fat and lean mass) (Kg), whole body bone mineral content (BMC) (g) and BMD (g/cm^2^) at lumbar spine (LS) L2-L4 and femoral neck (FN) were measured by DXA (Hologic QDR 4500; Hologic Inc., Waltham, MA, USA) before and 5 years after surgery. World Health Organization (WHO) criteria were used to defined osteoporosis (*T*-score below −2.5) and osteopenia (*T*-score between −1.0 and −2.5) [35]. Trabecular bone score (TBS) was calculated using LS DXA scans. As proposed by manufacturers MedImaps [36], we evaluated TBS in patients with BMI between 15 and 35 kg/m^2^. TBS ≥ 1.350 was considered normal; TBS between 1.200 and 1.350 as partially degraded and TBS ≤ 1.200 was defined as degraded [36]. The Spanish classic fracture risk assessment system (FRAX^®^) (Centre for Metabolic Bone Diseases, University of Sheffield, UK) corrected by TBS was used to evaluate the 10-year probability of hip fracture and major osteoporotic fractures [37,38]. High risk of major osteoporotic fractures was calculated including BMD and defined as a probability ≥7.5% or ≥5% and osteoporosis [39].

### 2.3. Standard Meal Test

A standard meal test (SMT) was performed before and 1 and 12 months after bariatric surgery. The SMT consisted of 200 mL of a liquid meal (Edanec^®^, NACE, Paris, France). Blood was drawn immediately before and 15, 30, 60 and 120 min following the SMT for GLP-1 and insulin determination. Fasting ghrelin, PYY and glucagon levels were measured before the SMT.

### 2.4. Laboratory Determinations

Phospho-calcium metabolism was determined before and 1 and 5 years after bariatric intervention. Glucose, calcium, phosphorus and alkaline phosphatase were determined using standard enzymatic methods. 25-hydroxyvitamin D (25(OH)D_3_) concentrations were determined using a radioimmunoassay (DiaSorin, Stillwater, MN, USA). Intact serum parathyroid hormone (PTH) was measured by a two-site immunoradiometric assay (Diagnostic System Laboratories, Webster, TX, USA). Plasma insulin was analyzed by immunoassay (Coat-A-Count Insulin, Diagnostic Products Corp., Los Angeles, CA, USA). GLP-1 was measured by radioimmunoassay (Millipore, Saint Charles, MO, USA) and plasma ghrelin by enzyme immunoassay (CUSABIO biotech, Wuhan, China). Glucagon and PYY were measured by enzyme immunoassay (Yanahaira Institute Inc., Awakura, Fujinomiya-shi Shizuoka, Japan).

### 2.5. Surgical Procedures

mRYGB combines both restriction (a small gastric pouch) and malabsorption (a 200 cm biliopancreatic limb with an alimentary limb of 100 cm). SG is a restrictive technique with a 75–80% of gastric volume reduction (stomach resection beginning 4 cm from the pylorus and ending at the angle of His). GCP is a restrictive and reversible procedure in which an invagination of the greater gastric curvature is performed instead of gastric resection.

### 2.6. Statistical Analysis

Based on preliminary data, the study design and sample size was calculated to detect a 20% difference in GLP-1 secretion (measured by the area under the curve (AUC) after (SMT) before and 1 year after bariatric surgery, with a power of 80% and α risk of 0.05 [33]. The primary outcome of the study was the predictive value of gut hormone dynamics (GLP-1, glucagon, PYY and ghrelin) on glucose metabolism improvement at 1 and 12 months after surgery for each procedure. A secondary outcome was the comparison of changes in BMD at 1 and 5 years between surgical techniques and their relationship with gastrointestinal hormones. There were no changes to trial outcomes after commencement of the study. Normally distributed variables were expressed as the mean (standard deviation) and non-normally distributed variables were expressed as the median (first and third quartile). Categorical variables were compared using Chi-square test and quantitative variables using ANOVA test for normally distributed variables and Kruskal–Wallis test was applied for non-normally distributed variables. GLP-1 area under the curve (AUC) was calculated by the trapezoidal method [40]. Two-way ANOVA with repeated measures was performed to analyze BMD changes throughout the observation period. Bivariate (Pearson or Spearman) and multivariate linear regression analyses and a mixed model were employed to determine associated and predicting factors of BMD decrease after bariatric surgery. Relevant clinical variables previously associated were included in the model (type of surgery, changes in weight, gastrointestinal hormones concentrations, phospho-calcium metabolism, metabolic parameters and the presence of T2D remission). Statistical analysis was performed using R software version 3.4.0 (R Foundation for Statistical Computing, Vienna, Austria). A *p*-value < 0.05 was considered statistically significant.

## 3. Results

Forty-five morbidly obese patients with T2D, aged 49.4 (7.8) years, BMI 39.4 (1.9) kg/m^2^, initial HbA_1c_ 7.7 (1.9) %, were consecutively randomized to mRYGB (*n* = 15), SG (*n* = 15), or GCP (*n* = 15) from May 2012 to February 2014. Follow up compliance was 97.78% (*n* = 44) at year 1 and 86.6% (*n* = 39) at year 5. Therefore, the 5-year evaluation was performed in patients undergoing mRYGB (*n* = 14), SG (*n* = 12) and GCP (*n* = 13); the rest of participants refused the BMD evaluation for personal reasons. Sixty-six percent of patients were women equally distributed between groups and menopause was present in 62% of those undergoing mRYGB, 60% in SG, and 75% in GCP, *p* = 0.704. Initial clinical, biochemical, and body composition characteristics were comparable between groups, except BMI, which was higher in GCP (Table 1). One-year outcomes and procedure complications, stratified according to the Clavien-Dindo classification, were previously described [33]. As a summary of earlier published data [27,33], at year one, TWL% was significantly greater in the mRYGB group compared to SG and GCP. At the end of the study, TWL% in the mRYGB group was −27.32 (7.87) vs. −18.00 (10.6) and −14.83 (7.84) in SG and GCP, respectively, *p* = 0.001. Regarding metabolic outcomes, at 5 year follow up, complete T2D remission was observed in 46.7% of patients undergoing mRYGB vs. 20.0% after SG and 6.6% after GCP, *p* < 0.001. Changes in biochemical parameters and body composition are shown in Table 2. Of note, mRYGB showed a better metabolic improvement and higher weight loss at an expense of fat mass. Serum calcium, phosphate and vitamin D levels were within normal concentrations and similar in all groups at the end of the study. PTH concentrations were slightly higher after mRYGB, but without reaching statistical significance (Table 2).

### 3.1. Changes in BMD after Bariatric Surgery

**Figure 1:**
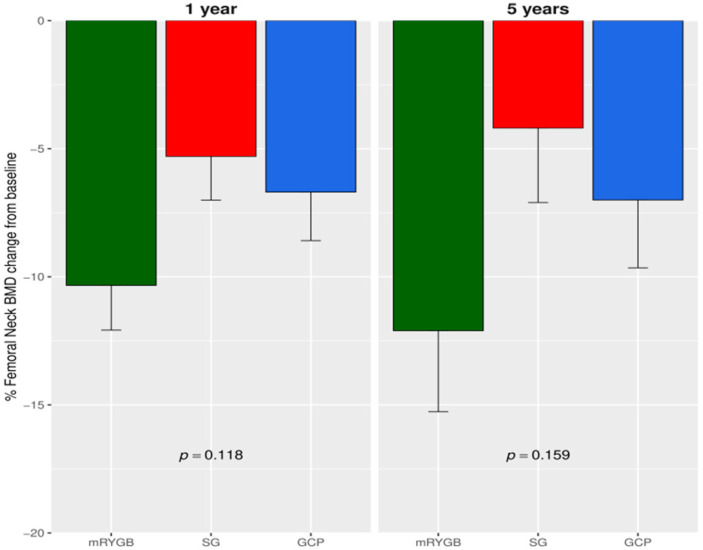
Percentage femoral neck BMD change at year 1 and 5 from baseline. BMD, bone mineral density; mRYGB, metabolic gastric bypass; SG, sleeve gastrectomy; GCP, greater curvature plication. *p* value result from comparing mRYGB with SG and GCP.

**Figure 2:**
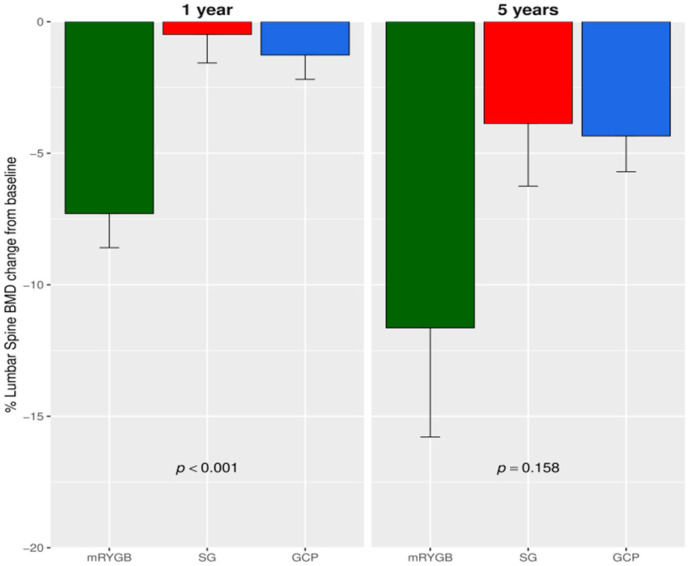
Percentage lumbar spine BMD change at year 1 and 5 from baseline. mRYGB, metabolic gastric bypass; SG, sleeve gastrectomy; GCP, greater curvature plication. *p*-value result from comparing mRYGB with SG and GCP.

From baseline to year one, a similar reduction in the FNBMD percentage was observed after mRYGB compared to SG and GCP: −10.34 (6.05) vs. −5.30 (6.17) vs. −6.69 (5.68), *p* = 0.118. However, a greater decrease at LS BMD percentage was detected after mRYGB compared to SG and GCP: −7.29 (4.6) vs. −0.48 (3.9) vs. −1.2 (2.7), *p* < 0.001). The overall percentage descent from baseline to year five at the FN was −12.10 (11) vs. −4.19 (10) vs. −7.0 (7.96), *p* = 0.159 (Figure 1) and at LS: −11.64 (15.0) vs. −3.87 (7.91) vs. −4.34 (4.07), *p* = 0.158 (Figure 2). Thus, BMD at LS was significantly lower at 5 years after mRYGB (Table 2). We performed a two-way ANOVA analysis with repeated measures. At FN, no differences were observed between surgical techniques. Only after mRYGB, we observed effect of time that was significant between baseline and one year (*p* < 0.001), but not between one to 5 years, indicating that changes at this site took place during the first year after mRYGB. Regarding LS, the mixed model found significant differences in mRYGB compared to SG and GCP; and only after mRYGB was the effect of time significant in each observation period, *p* < 0.001, indicating an ongoing process along the 5 year follow-up.

At the end of the study, FN osteopenia was present in 50.0% (*n* = 7) after mRYGB, 25.0% (*n* = 3) in SG and 25.0% (*n* = 3) in GCP participants; while osteoporosis only affected one patient in the GCP group, *p* = 0.365. At LS, osteopenia was present in 78.6% (*n* = 11) of the mRYGB group vs. 33.3% (*n* = 4) and 50.0% (*n* = 6) in SG and GCP, respectively, and osteoporosis affected two patients (one in each mRYGB and GCP groups), *p* = 0.030. No bone fractures were observed during the study. At year 5, TBS values did not show statistical differences when comparing mRYGB with SG and GCP: 1.288 (0.09) vs. 1.320 (0.11) vs. 1.311 (0.12), *p* = 0.759. However, 85% of patients had partially or totally degraded microarchitecture after mRYGB compared to 66.7% after SG and 58.3% in GCP, without reaching significant differences among groups, *p* = 0.291. The ten year risk of major osteoporotic fracture was 2.5% (1.20) in mRYGB vs. 2.1% (1.28) and 2.6% (1.50) in SG and GCP, respectively, *p* = 0.74. Risk of hip fracture was 0.30% (0.20) in mRYGB vs. 0.20% (0.56) in SG and 0.10% (0.63) in GCP, *p* = 0.995.

### 3.2. Correlation of BMD with Anthropometrics, Biochemical and Hormonal Parameters

Bivariate correlations between BMD changes at FN and LS with body composition, biochemical parameters and hormonal changes after surgery are shown in Table 3.

When analyzing variables associated with the reduction in BMD after surgery, the decrease at FN and LS correlated positively with reduction in body weight, fat mass, lean mass and HbA_1c_ values at 5 year follow-up. Additionally, BMD decline at LS correlated inversely with alkaline phosphatase and the increase from baselines to one year in osteocalcin and the AUC for GLP-1. No correlations were found between BMD changes and phospho-calcium parameters at the fifth year nor with other gastrointestinal hormones.

### 3.3. Changes in BMD Regarding 5 Year T2D Outcomes after Surgery

Considering the possible influence of metabolic effects in skeleton metabolism, we compared BMD changes in patients with persistent T2D remission vs. non-remitters five years after surgery. BMD reduction was greater among remitters vs. non-remitters. At FN, the percentage of reduction was −4.37 (9.90) in non-remitters, −16.08 (1.98) in partial remitters and −10.57 (10.7) in complete remitters, *p* = 0.042. At LS, the percentage of reduction was −2.12 (10.7) in non-remitters, −16.15 (6.57) in partial remitters and −11.97 (8.01) in complete remitters, *p* = 0.005. No significant differences were observed in TBS values regarding T2D remission. Of note, no significant differences between groups were observed in antidiabetic agents used before surgery. Patients requiring pharmacological treatment after surgery were mainly treated with metformin and DPP-IV inhibitors.

### 3.4. Predicting Factors of BMD Reduction after Surgery

We performed a multiple regression analysis to better determinate BMD predictors. Weight at 5 year was found to be the only variable that predicted BMD changes at FN (adjusted *R*-squared: 0.3218, *p*-value: 0.00247). On the other hand, the type of surgery (mRYGB) and menopause were the variables that predicted BMD changes at LS (adjusted *R*-squared for the model: 0.2507, *p* < 0.005. Other variables such as changes in HbA_1c_, phospho-calcium parameters and gastro-intestinal hormones including GLP-1 AUC were not final predictors of bone outcomes at either location. Coefficients of the regression model are shown in Table 4. We completed the analysis with a mixed model obtaining similar results; with this model, the effect of time was also significant, in agreement with the results of two-way ANOVA analysis.

## 4. Discussion

To our knowledge, this is the first study that has compared 5 year BMD outcomes between three different bariatric procedures (mRYGB, SG, and GCP) in the setting of a RCT. We found that mRYGB, characterized by a hypoabsortive component, showed a greater deleterious effect on LS at long-term compared to SG and GCP. Women with menopause had the greatest risk of bone loss at LS.

Bariatric surgery produces detrimental effects on bone health and there is a significant and non-uniform reduction in BMD across different bone sites [3,16,41,42]. In the short-term, the preferential bone loss at FN and weight-bearing sites suggests that this could be a response to unloading after weight loss [43,44]. Only a few previous studies, mainly focusing on standard RYGB, have evaluated long-term BMD outcomes. In this sense, two observational studies have reported bone deterioration 5 years after RYGB. Raoof et al. [45] found a linear and significant decline in BMD at FN (25%) and LS (19%) among 32 women that had not received calcium and vitamin D supplementation. Lindeman et al. [46] also detected a greater reduction in BMD at total hip (15.3%) and less reduction at LS (7.8%), although the majority of bone loss occurred within the first 2 years. Recently, Hansen et al. analyzed BMD changes 7 years after RYGB [47]. Among 17 participants, a BMD decline of 17% at total hip and 8% at LS was observed. Changes at LS occurred during the first 2 years, although there was a continuous decline in total hip BMD between the second and seventh year after surgery. In our cohort, we observed an overall reduction around 12% after mRYGB (FN and LS) and between 4 and 7% (FN) and 3.8 and 4.3% (LS) after restrictive procedures. FN BMD loss was more pronounced during the first year after surgery when maximum weight loss was achieved, probably due to the effects of skeletal unloading. On the other hand, LSBMD decline was not as pronounced during the first year but it was an ongoing process, especially after mRYGB. The differences observed when compared to former studies could be explained by the heterogeneity in the patient’s characteristics (proportion of women, menopausal status), calcium and vitamin D supplementation and type of surgery; particularly in our cohort where mRYGB with a greater hypoabsortive component was performed.

There is a lack of studies comparing long-term BMD outcomes after different surgical procedures. In a meta-analysis, comparing BMD changes after RYGB and SG, bone outcomes were similar [48]. However, in only one of 13 studies included, the follow-up time was greater than 2 years. The STAMPEDE study compared bone changes after RYGB and SG versus intensive medical treatment in patients with T2D in the setting of a RCT. At 2 years, BMD changes were similar between groups, but at 5 years, RYGB showed a greater increase in bone metabolism markers compared to SG, thus supporting our findings [23,49]. No previous data have been published analyzing BMD changes after GCP.

Bone loss after bariatric surgery is complex and many predicting factors have been proposed. Changes like weight loss, especially at weight bearing sites [50], and the lean mass decline have been associated to the BMD reduction [51,52]. We found a positive correlation between whole BMD, FN and LS, 5 years after surgery with final weight; fat mass and lean mass. Our results therefore support the influence of body composition variations on BMD changes after bariatric surgery and highlight the importance of preserving lean mass to reduce the risk of osteosarcopenia [53]. Interestingly, in the multiple regression analysis, FN decline was influenced mainly by weight loss, supporting the hypothesis of a direct effect of weight unloading. However, at LS, other predicting factors such as menopause and hypoabsortive techniques were found. In this sense, it has been suggested that LS trabecular bone is metabolically more active and therefore more reactive to hormonal changes [54]. Moreover, the peripheral conversion of androgens to estrogens by adipose aromatase can be compromised with body fat reduction and this could negatively influence LS BMD, mainly in older menopausal women [24]. Also, body fat secretes many adipokines, such as leptin and adiponectin, which have been implicated in bone metabolism [21,55].

Micronutrient absorption is commonly affected after bariatric surgery. Significantly lower vitamin D and higher PTH levels have been reported in surgically treated obese patients compared to nonsurgical obese patients [52]. Carrasco et al. [56] reported a similar calcium reduction in both SG and RYGB 2 years after surgery compared to baseline, and it was not associated with changes in BMD. Our findings go in the same direction, and we did not find a relationship between 5 year postsurgical levels of calcium, vitamin D, PTH or PTH variation from the baseline values with BMD reduction. However, we should consider that our patients were given proper calcium and vitamin D supplementation and normal mean values of calcium and vitamin D were maintained across the study. However, as mRYGB has a greater malabsortive component compared to classic RYGB, we cannot discard deficiencies in other micronutrients and minerals that could affect bone health.

The influence of gastrointestinal hormone changes after bariatric surgery in bone metabolism is still unclear. While some studies in mice suggest that incretins like GLP-1 and GIP may have a beneficial effect on bone [18,57], a negative association between bone formation markers and PYY has been reported among adolescents with anorexia nervosa [58]. Carrasco et al. [21] observed that ghrelin reduction was associated with BMD loss after RYGB and SG. Another study in patients with obesity and T2D showed that fluctuation in a ghrelin gene product (unacylated ghrelin) after RYGB was associated with the reduction in BMD [59]. In our cohort, as we described previously [33], we observed a significantly higher increase in the AUC for GLP-1, as well as fasting values of PYY and ghrelin one year after mRYGB compared to restrictive procedures. At the fifth year, we found a negative correlation between BMD reductions at LS with an increase in AUC for GLP-1 observed one year after surgery. This can be explained by the fact that the patients undergoing RYGB who experienced the greatest LS loss showed the greatest increase in AUC for GLP-1. We also found correlation between whole BMD changes at the end of the study with other hormones one year after surgery, such as ghrelin (negative), and glucagon and insulin (positive). Nevertheless, in the multiple regression analysis, gastrointestinal hormones, particularly GLP-1, lost statistical significance, casting doubts on their key role on BMD changes after bariatric surgery.

Recently, BMD has been related to glucose tolerance status [60]. It has been suggested that chronic hyperglycaemia could degrade bone quality through the inhibition of osteocalcin, increased reactive oxygen species, bone accumulation of advanced glycation end products or the inhibition of GLP-1 [40]. In a cross-sectional matched cohort study including individuals with BMI > 35kg/m^2^ and T2D (treated by RYGB and non-operated), the authors did not observe a relation between T2D status at the end of the study (remission vs. non-remission) with bone loss [61]. Conversely, in our study, LSBMD at 5 year follow-up correlated positively with HbA_1c_ values and we found a significantly lower BMD at FN as well as at LS among T2D remitters compared to non-remitters. However, although a better bone quality could be expected in T2D remitters, similar bone microarchitecture measured with TBS values was observed in remitters and non-remitters. Probably, the fact that patients undergoing RYGB were those with greater T2D remission and greater weight loss could explain our findings. Also, we cannot underestimate the effect of oral antidiabetic agents on bone. However, no significant differences in anti-diabetic agents were observed at the beginning of the study. Those patients where T2D persisted or recurred after surgery were mostly treated with metformin and DPP-IV inhibitors. No patient received glitazones or SGLT-2 that might negatively affect bone.

Surgically induced weight loss is associated with an increased risk of vertebral and non-vertebral fractures that starts in the second year, but becomes significant at the fifth year after surgery [62,63]. In a meta-analysis, the highest possibility of fracture was found after malabsortive procedures (biliopancreatic diversion) followed by the mixed techniques (RYGB) without an increased risk after restrictive procedures (adjustable gastric banding and SG) compared to the nonsurgical population [64]. Recently, the 26 year results of the S.O.S study [65] observed the highest incidence rate for first fracture after RYGB compared to restrictive procedures. In our cohort, the small size and time of observation were probably the reason why we had no bone fractures. Nonetheless, at 5 years, we observed a high percentage of patients with osteopenia, reaching 70% at LS after mRYGB, but a low percentage of osteoporosis. Of note, mean Z-scores (which compare BMD with same-aged healthy population) were below 0 after mRYGB but no patient was ≤−2, the threshold for BMD below the expected range for the age. It is also unsettling that the fracture risk calculated with the FRAX algorithm was low in our patients and none of our patients fulfilled the criteria for treatment. The fact that age is the factor that counts the most in the algorithm along with previous fractures, and that our patients were relatively young, can probably explain the low values obtained. Nevertheless, in our cohort, we analyzed TBS which is a simple, non-invasive and inexpensive method to assess bone microarchitecture. To date, very few studies have evaluated TBS after bariatric surgery [42,66]. In a previous study reported by us including 38 obese women with an initially normal TBS score, 26.3% of patients had abnormal TBS values 3 years after RYGB [67]. In the present cohort, abnormal TBS at 5 years (partial or totally degraded) reached 85% of patients after mRYGB, but also about 50% of those undergoing restrictive procedures. This supports the hypothesis of a continuous bone microarchitecture declining over time which could increase the risk of fracture in the longer term.

The findings of our study cannot be generalized to all patients undergoing bariatric surgery as only three types of procedure were analyzed in a selected population with T2D. Our results should be understood taking into consideration several limitations. Firstly, the size of the study group is small and the diversity of participants (gender, menopause status, metabolic improvement after surgery) could influence the final BMD. In addition, we cannot discard the effect of antidiabetic drugs on bone. Secondly, the DEXA scan has limited accuracy in obese individuals because of the excess fat overlying bone and the heterogeneity of its distribution [68]. Also, BMD at LS can be altered by the presence of degenerative disk disease and osteophytes which can lead to falsely elevated measurements. Lastly, the small number of gastrointestinal hormones determined that were only evaluated during the first year after bariatric surgery.

## 5. Conclusions

In summary, our results show that mRYGB induces higher deleterious effects, especially at LS compared to SG and GCP. Elderly women with menopause undergoing mRYGB are at a higher risk of bone deterioration. Phospho-calcium metabolism and gastrointestinal hormone changes do not seem to have a major role in BMD outcomes 5 years later. Our findings reinforce the importance of long-life bone surveillance in bariatric patients and the need to select the bariatric technique according to the patient’s risk for fractures. Restrictive procedures should be preferable over mRYGB, especially in those who are susceptible to osteoporosis.

## Figures and Tables

**Table 1:** Patient’s baseline characteristics.

Parameter	Metabolic Gastric Bypass	Sleeve Gastrectomy	Greater Curvature Plication	*p*
Sex (male/female)	7/8	5/10	3/12	0.301
Age (years)	51.1 (7.70)	49.2 (9.16)	49.7 (8.12)	0.827
Weight (kg)	103.01 (10.8)	102.30 (10.7)	105.53 (11.8)	0.301
BMI (kg/m^2^)	38.73 (2.01)	39.02 (1.68)	40.90 (1.44)	0.004 *
HbA_1c_ (%)	7.39 (1.95)	7.89 (1.71)	8.05 (2.15)	0.498
Calcium (mmol/L)	2.35 (0.12)	2.37 (0.12)	2.6 (0.12)	0.978
Phosphate (mmol/L)	1.06 (0.16)	1.09 (0.18)	1.08 (0.15)	0.856
PTH (pmol/L)	4.75 (4.46)	3.66 (1.58)	5.05 (4.45)	0.803
Vitamin D (nmol/L)	54.99 (21.35)	52.67 (29.78)	52.78 (25.97	0.606
Fat Mass (kg)	36.53 (8.09)	34.22 (5.57)	35.01 (12.27)	0.414
Lean Mass (kg)	57.39 (10.80)	53.78 (8.29)	50.82 (17.33)	0.670
FNBMD	0.89 [0.84;0.96]	0.90 [0.81;0.96]	0.95 [0.84;1.07]	0.344
FN T-score	−0.05 [−0.50;0.40]	0.08 [−0.40;0.50]	0.85 [−0.25;1.55]	0.077
FN Z-score	0.81 [0.48;1.37]	0.96 [0.10;1.60]	1.54 [0.73;2.00]	0.134
LSBMD	1.03 [0.98;1.09]	1.11 [1.04;1.21]	1.08 [0.99;1.14]	0.255
LS T-score	−0.59 [−1.15;0.05]	0.28 [−0.40;1.10]	0.09 [−0.45;0.50]	0.082
LS Z-score	0.01 [−0.50;0.50]	0.79 [0.10;1.80]	0.62 [−0.05;1.03]	0.239

Data are expressed as mean (standard deviation) for normal distributed variables and median (first and third quartiles) for non-normal distributed variables. BMD, bone mineral density; BMI, body mass index; FN, femoral neck; HbA_1c_, glycated hemoglobin, LS, lumbar spine; *p*, statistical significance; PTH, parathyroid hormone; *, *p* < 0.05 was considered statistically significant.

**Table 2:** Patient’s characteristics 5 years after bariatric surgery.

Parameter	Metabolic Gastric Bypass	Sleeve Gastrectomy	Greater Curvature Plication	*p*
Sex (male/female)	6/8	4/8	3/10	0.552
Age (years)	55.2 (7.40)	55.2 (8.30)	53.6 (8.54)	0.700
Weight (kg)	74.7 (9.97)	84.4 (17.0)	89.2 (11.7)	0.014 *
BMI (kg/m^2^)	28.1(2.99)	32.0 (4.56)	34.7 (3.68)	<0.001 *
HbA_1c_ (%)	5.43 (0.69)	6.97 (1.32)	7.07 (1.66)	0.002 *
Calcium (mmol/L)	2.32 (0.10)	2.38 (0.10)	2.38 (0.10)	0.221
Phosphate (mmol/L)	1.13 (0.21)	1.08 (0.22)	1.08 (0.15)	0.722
PTH (pmol/L)	8.39 (3.50)	5.66 (2.19)	6.73 (2.63)	0.059
Vitamin D (nmol/L)	61.9 (46.7)	65.3 (33.6)	73.4 (52.3)	0.801
Fat Mass (kg)	31.2 (6.37)	40.0 (8.82)	41.9 (6.86)	0.001 *
Lean Mass (kg)	39.4 (6.74)	43.4 (8.91)	43.1 (7.98)	0.352
FNBMD	0.77 [0.72;0.82]	0.83 [0.78;0.92]	0.85 [0.74;0.98]	0.259
FN T-score	−1.08 [−1.68;−0.80]	−0.50 [−0.92;0.23]	−0.40 [−1.07;0.38]	0.186
FN Z-score	−0.08 [−0.40;0.20]	0.60 [−0.05;1.15]	0.78 [0.00;1.40]	0.081
LSBMD	0.89 [0.82;0.94]	1.04 [0.91;1.16]	0.99 [0.89;1.12]	0.020 *
LS T-score	−1.55 [−2.05;−1.20]	−0.04 [−1.12;1.21]	−0.83 [−1.68;0.23]	0.011 *
LS Z-score	−0.82 [−1.30;−0.40]	0.93 [0.15;1.83]	0.34 [−0.80;1.40]	0.004 *

Data are expressed as mean (standard deviation) for normal distributed variables and median [first and third quartiles]) for non-normal distributed variables. BMD, bone mineral density; BMI, body mass index; FN, femoral neck; HbA_1c_, glycated hemoglobin, LS, lumbar spine; *p*, statistical significance; PTH, parathyroid hormone; *, *p* < 0.05 was considered statistically significant.

**Table 3:** Correlations of BMD changes at the femoral neck and lumbar spine with body composition, biochemical parameters and hormonal changes after surgery.

Characteristic	ΔFN BMD		ΔLS BMD	
	*R*	*p*-Value	*R*	*p*-Value
Weight_b_ (kg)	0.450	0.009 *	0.507	0.003 *
BMI_b_	0.104	0.563	0.061	0.733
Weight_5_ (kg)	0.661	<0.001 *	0.656	<0.001 *
BMI_5_	0.588	<0.001 *	0.499	0.003 *
Fat mass_5_ (kg)	0.596	<0.001 *	0.509	0.003 *
Lean mass_5_ (kg)	0.408	0.021 *	0.565	<0.001 *
Vitamin D_5_ (nmol/L)	−0.58	0.753	0.009	0.963
PTH_5_ (pmol/L)	−0.224	0.217	−0.251	0.166
APh_5_ (µkat/L)	−0.260	0.143	−0.418	0.016 *
ΔOsteocalcin_b−1a_ (µg/L)	−0.241	0.191	−0.360	0.047 *
HbA_1c5_ (%)	0.452	0.008 *	0.495	0.003 *
ΔGLP-1AUC_b−1a_	−0.337	0.080	−0.528	0.004 *
ΔPYY_b−1a_	−0.114	0.586	−0.124	0.553
ΔGlucagon_b−1a_	−0.096	0.646	0.045	0.830
ΔGhrelin_b−1a_	0.142	0.453	0.241	0.199
ΔAUC Insulin_b−1a_	−0.110	0.547	0.116	0.553

Spearman and Pearson analysis were performed. APh, alkaline phosphatase; AUC, area under the curve; BMD, bone mineral density; FN, femoral neck; GLP-1, glucagon like-peptide 1; HbA_1c_, glycated hemoglobin; LS, lumbar spine; PTH, parathyroid hormone; PYY, peptide YY; b, baseline; 5, values at 5 years; Δ changes from baselines to 5 years; Δ b−1, changes from baselines to year 1; *, *p* < 0.05 was considered statistically significant.

**Table 4:** Coefficients of the regression model.

**ΔFN BMD**	**Estimate**	**Std. Error**	***p*-Value**
(Intercept)	−40.439	8.301	<0.001 *
mRYGB	(1 Ref.)		
SG	3.584	3.636	0.333
GCP	−0.559	4.028	0.891
weight	0.379	0.106	<0.001 *
**ΔLS BMD**	**Estimate**	**Std. Error**	***p*-Value**
Intercept	−5.825	3.268	0.086
mRYGB	(Ref.)		
SG	9.113	4.005	0.031
GCP	9.881	4.228	0.027
Male	(Ref.)		
Female No Menop	−9.916	4.660	0.042
Female Menop	−11.463	3.831	0.006

BMD, bone mineral density; FN, femoral neck; GCP, greater curvature plication; LS, lumbar spine; mRYGB, metabolic gastric bypass; SG, sleeve gastrectomy; Menop, menopause; Δ, changes from baseline to year 5, *, *p* < 0.05 was considered statistically significant.
